# Case report: Chemotherapy plus sintilimab for the treatment of gastroesophageal junction hepatoid adenocarcinoma with liver metastasis: a case study with literature review

**DOI:** 10.3389/fimmu.2025.1513604

**Published:** 2025-01-27

**Authors:** Genlin Lu, Jiarui Tu, Jinming Tu, Renya Jiang

**Affiliations:** ^1^ Department of General Surgery (Key Disciplines of Medicine in Quzhou City), Longyou County People’s, Longyou People’s Hospital Affiliated with Sir Run Run Shaw Hospital, Zhejiang University School of Medicine, Quzhou, China; ^2^ Clinical Medicine Department, Tongji Medical College, Huazhong University of Science and Technology, Wuhan, China; ^3^ Department of Gastroenterology, Longyou County People’s Hospital, Longyou People’s Hospital Affiliated with Sir Run Run Shaw Hospital, Zhejiang University School of Medicine, Quzhou, China; ^4^ Department of Hepatobiliary Surgery, Quzhou City People’s Hospital, Quzhou, China

**Keywords:** gastroesophageal junction, hepatoid adenocarcinoma, chemotherapy, sintilimab, liver metastasis

## Abstract

**Objective:**

To elucidate the clinicopathological features and treatment of metastatic gastroesophageal junction hepatoid adenocarcinoma (GEJ HAC)using a case study and literature review.

**Methods:**

Clinical presentation, results of histology and immunohistochemistry, and next-generation sequencing(NGS) in a patient with GEJ HAC metastasizing to the liver were reviewed. Chemotherapy (SOX or S-1) plus sintilimab was administered.

**Results:**

A 65-year-old male patient with a history of hypertension was admitted to the hospital due to a one-week increase in serum AFP levels. There was a small intraluminal mass at the GEJ and a metastatic lesion in liver segment VIII, as well as enlarged perigastric and retroperitoneal lymph nodes. Tumor cells in both the GEJ and liver tissue exhibited a glandular shape with a nest-like adenoid structure. Immunohistochemical (IHC) analysis of the GEJ tissue showed positivity for AFP, CA19-9, CK7, CK20, MUC-1, P53 (wild type), Glypican-3, and HepPar-1, and negativity for Arginase-1, CD10, and Her-2. In the metastatic liver tissue, IHC testing demonstrated positivity for AFP, CD10, CK19, CK20, HepPar-1, MUC-1, Ki-67, and P53 (wild type), while CK7 was negative. The NGS report of GEJ mass indicated that the *JAK2* and *TP53* genes harbored missense mutations, while the *MLH1, MSH2, MSH6, PMS2, ERBB2, EGFR, PIK3CA, APC, CTNNB1, CDH1, and DPYD* genes were normal. The patient’s serum levels of CEA, CA19-9, and AFP were sharply decreased. The patient achieved a major pathological response (MPR) and remains in a progression-free stage.

**Conclusions:**

Sintilimab-based chemotherapy has proven efficacy in achieving a MPR and maintaining a progression-free state for a patient with GEJ HAC that has metastasized to the liver.

## 1 Introduction

Gastroesophageal junction(GEJ) adenocarcinoma is one of the deadliest cancers worldwide (1). Hepatoid adenocarcinoma (HAC), a highly aggressive, metastatic, and potentially chemosensitive tumor, is characterized by aberrant hepatocellular differentiation occurring in extrahepatic organs, such as the stomach (2). Surgery combined with adjuvant therapy has improved clinical outcomes for GEJ HAC (3–7) (**Table 1**). However, others believe that treatment options for GEJ HAC are limited and the prognosis remains very poor (8–10) (**Table 1**).

**Table 1:** Summary of publications on gastroesophageal junction hepatoid adenocarcinoma.

First author year	Number of patients	Age	Gender	Treatment	Survival	Clinical stage	Serum AFP level (ng/mL)
Nagai Y 2014 (3)	1	62	male	total gastrectomy and lower esophagus resection plus chemotherapy(S-1 and cisplatin)	alive at last FU	TXNXM1	higher than normal
de Castria TB 2021 (8)	1	62	male	trastuzumab plus FOLFOX in the first line and paclitaxel and ramucirumab in the second line	alive at last FU	NR	19568
Apostolou K 2020 (4)	1	35	male	Ivor Lewis esophagectomy with TACE	59 weeks	T3 N0 M0	normal
Gálvez-Muñoz E 2009 (5)	1	75	male	pallitive total gastrectomy with chemotherapy (cisplatin and capecitabine)	32 weeks	TXNXM1	4500
Ye MF 2013(6)	3	58,54,61	two males One female	total gastrectomy(one patient), an expanded gastrectomy (another patient),chemotherapy(all)	32-144 weeks	pT2N0M1,pT2N0M0,NR	5845,858.1,>50000
Tazuma S 2020 (7)	1	51	male	total gastrectomy with chemotherapy (S-1 and wPTX/RAM)	16 weeks	T3 N0 M0	higher than normal
Yazdanpanah O 2024 (9)	1	35	male	Flot	51 weeks	NR	61395
Sarmast N 2019 (10)	1	53	male	sorafenib	2 weeks	NR	3861

Flot, docetaxel, oxaliplatin, leucovorin and 5-fluorouracil; TACE, trans-arterial chemoembolization; FOLFOX, 5-fluorouracil, oxaliplatin and leucovorin; NR, no report; FU, follow-up.

Sintilimab, a fully recombinant human IgG4 anti-PD-1 monoclonal antibody, binds to the PD-1 receptor and blocks its interaction with PD-L1 and PD-L2, demonstrating a robust anti-tumor effect. The ChiCTR1900024428 study suggested that sintilimab in combination with concurrent chemoradiotherapy exhibited promising efficacy and a favorable safety profile in the perioperative treatment of locally advanced gastric (G)/GEJ adenocarcinomas (11). The NCT02937116 study demonstrated that sintilimab plus CapeOx, as a first-line treatment for locally advanced or metastatic G/GEJ adenocarcinoma, shows acceptable safety and promising efficacy (12). Currently, there is limited evidence to demonstrate whether patients with GEJ HAC metastatic to the liver can benefit from sintilimab-based chemotherapy. This case report aims to elucidate the clinicopathological features and treatment of metastatic GEJ hepatoid adenocarcinoma through a clinical case study and literature review.

This article is presented in accordance with the CARE reporting checklist.

## 2 Case presentation

### 2.1 General information

On April 17, 2023, a 65-year-old man was admitted to Longyou County People’s Hospital due to an elevated serum AFP level. During a physical examination at a community hospital a week ago, his serum AFP level was found to be 4887.13 ng/mL (reference range: <8.78 ng/mL). He exhibited no symptoms such as nausea, vomiting, diarrhea, abdominal pain, abdominal distension, hematochezia, hematemesis, fatigue, poor appetite, skin ecchymosis, oliguria, gingival bleeding, or nasal bleeding. He was currently taking 2.5 mg of amlodipine besylate daily for the treatment of hypertension that had lasted for more than 10 years. No risk factors for hepatocellular carcinoma, such as a history of alcohol consumption, known liver disease, or viral hepatitis, were reported. He had no history of smoking, psychosocial, oncological, or genetic diseases, nor any relevant family history.

Upon physical examination, no enlarged neck lymph nodes, supraclavicular lymph nodes, jaundice, varicose veins on the abdominal wall, tenderness, rebound tenderness, hepatomegaly, splenomegaly, or shifting dullness were found. His BMI was 22.64 kg/m².

**Figure 1:**
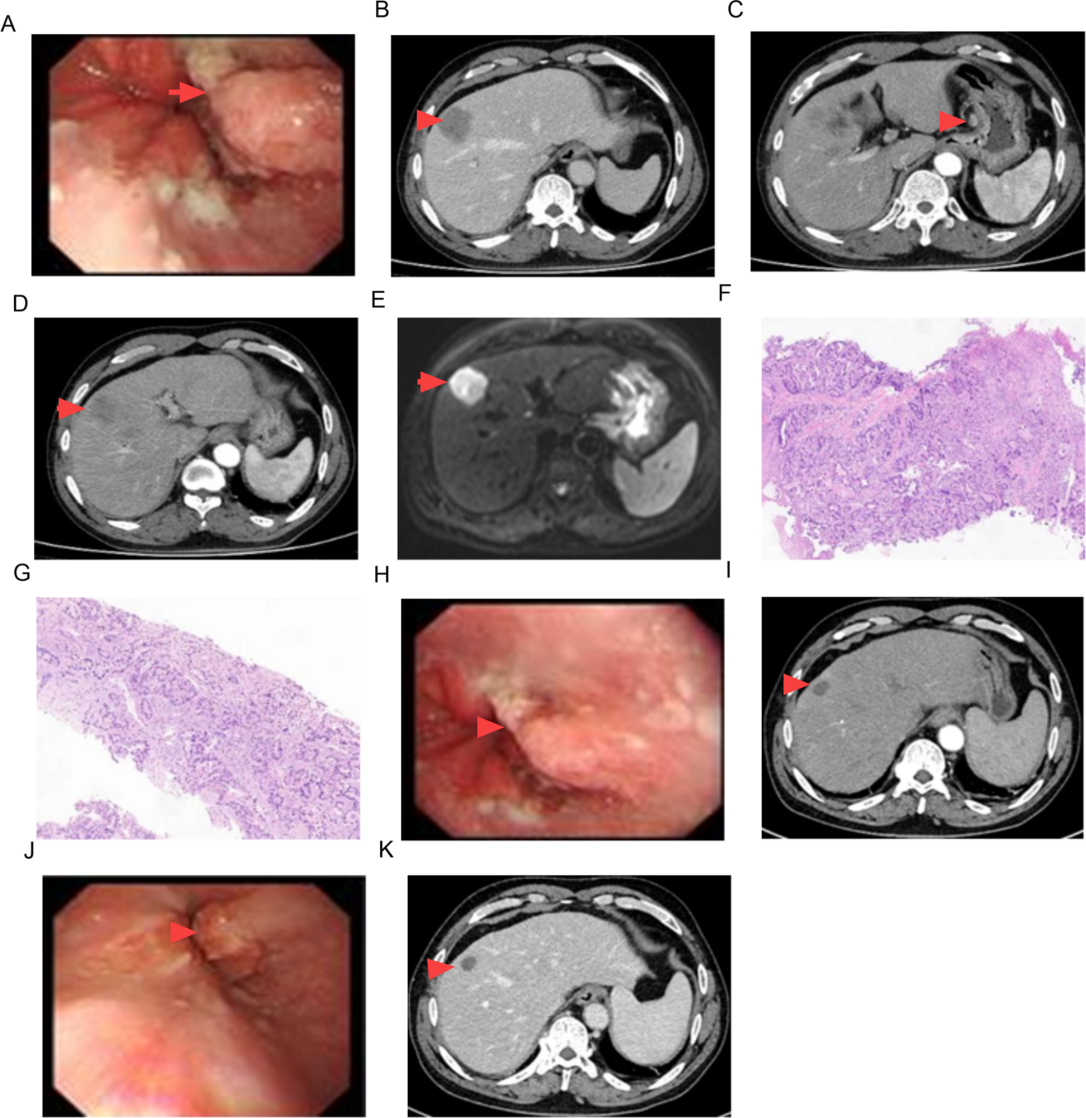
Imaging findings of CT, MRI, pathology and endoscopy. GEJ mass [**(A)** 0 month, **(H)** 6 months, **(J)** 12 months)], **(B–D)** liver metastasis and enlarged lymph nodes in CT imaging, **(E)** liver metastasis in MRI imaging, **(F)** pathologic findings of GEJ mass (HE staining × 100), **(G)** pathologic findings of liver metastasis (HE staining × 100), **(I)** CT scan in 6 months, **(K)** CT scan in 12 months.

**Figure 2:**
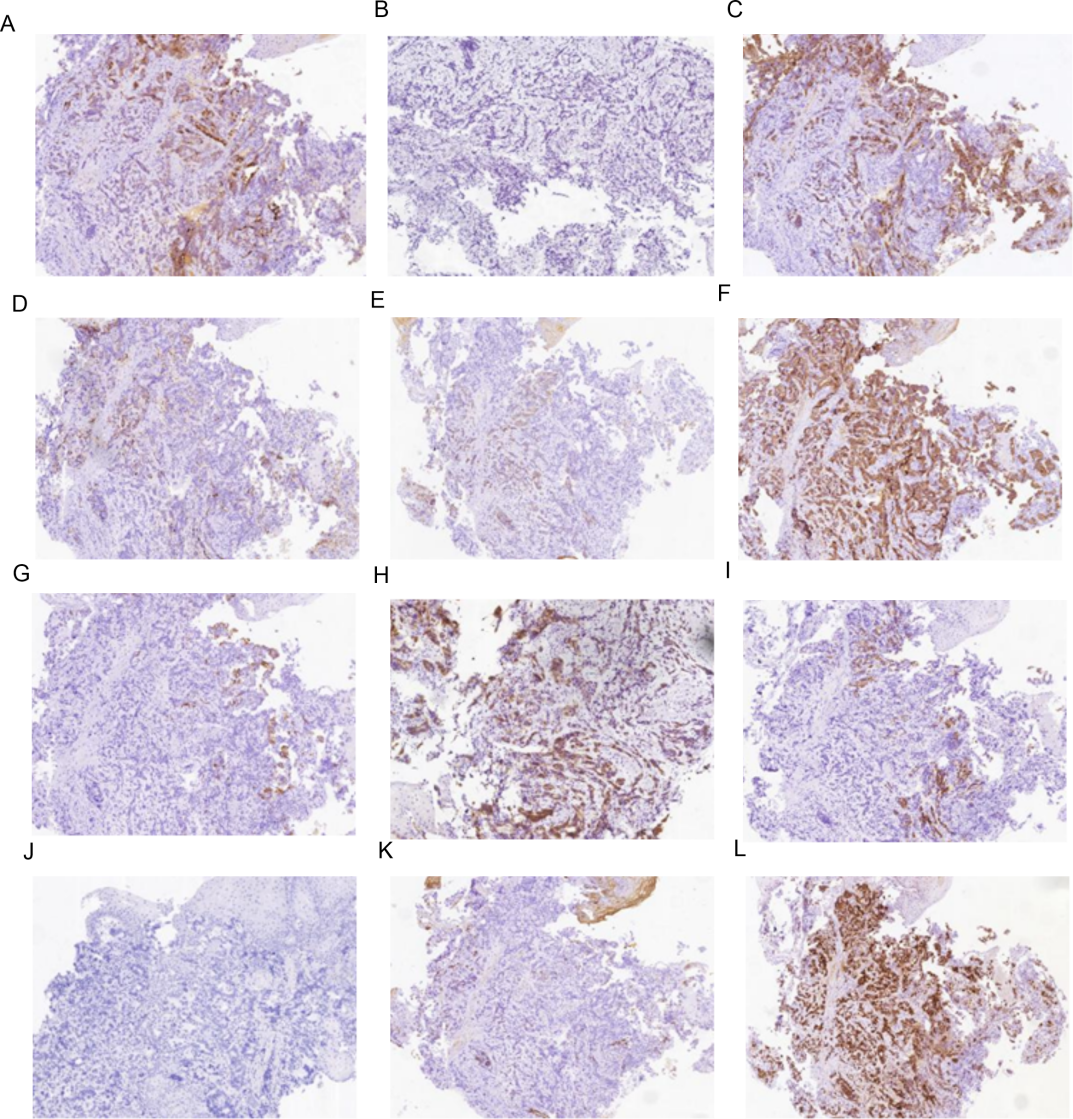
Immunohistochemistry results of mass at GEJ. **(A)** AFP positive (IHC [EnVision] 100×), **(B)** Arginase-1 negative (IHC [EnVision] 100×), **(C)** CA199 positive (IHC [EnVision] 100×), **(D)** CD10 negative (IHC [EnVision] 100×), **(E)** CK7 positive (IHC [EnVision] 100×), **(F)** CK19 positive (IHC [EnVision] 100×), **(G)** CK20 positive (IHC [EnVision] 100×), **(H)** Glypican-3 positive (IHC [EnVision] 100×), **(I)** HepPar-1 positive (IHC [EnVision] 100×), **(J)** Her-2 negative (IHC [EnVision] 100×), **(K)** MUC-1 positive (IHC [EnVision] 100×), **(L)** P53(wild type)positive (IHC [EnVision] 100×).

**Figure 3:**
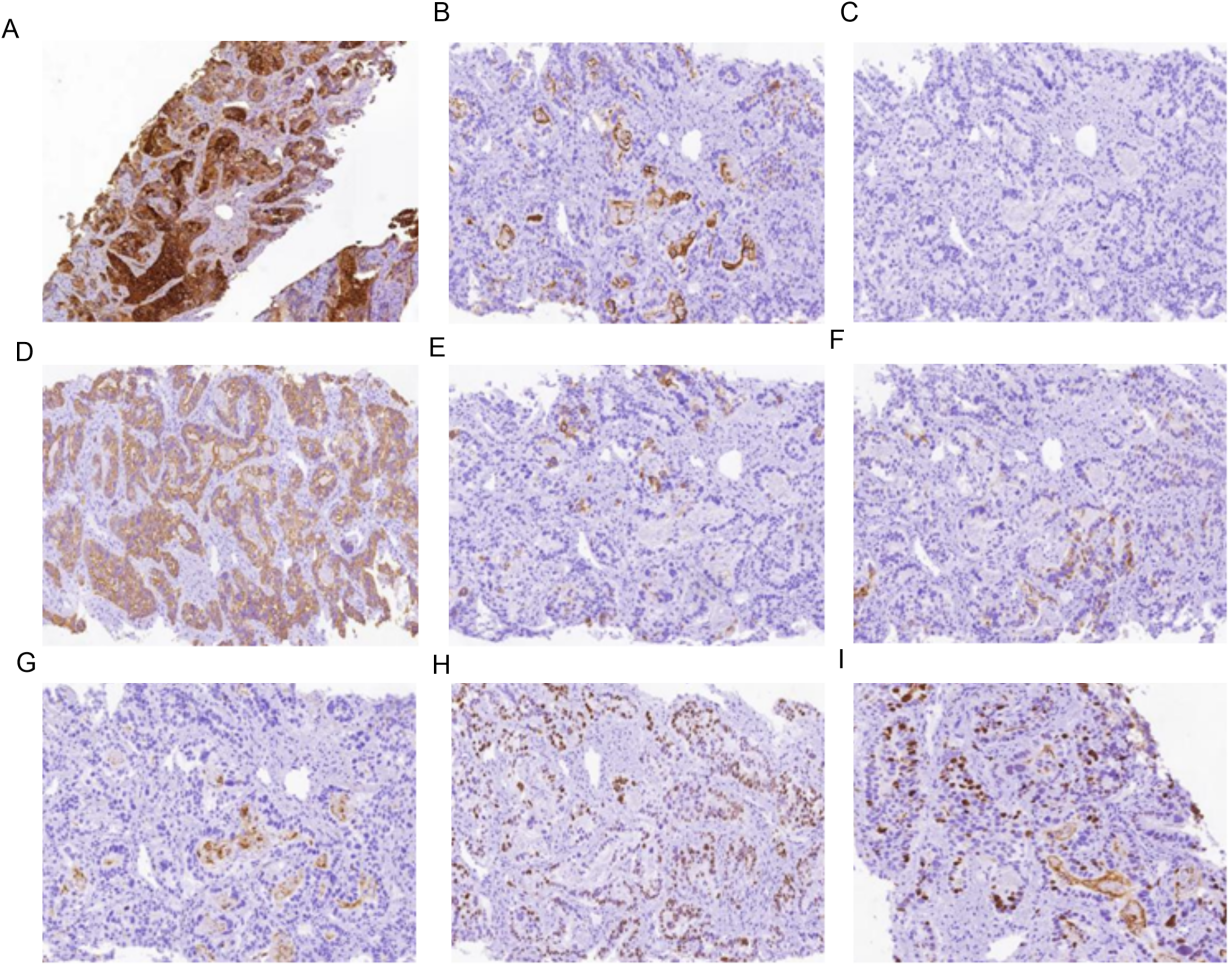
Immunohistochemistry results of metastatic liver tissue **(A)** AFP positive (IHC [EnVision] 100×) **(B)** CD10 positive (IHC [EnVision] 100×) **(C)** CK7 negative (IHC [EnVision] 100×) **(D)** CK19 positive (IHC [EnVision] 100×) **(E)** CK20 positive (IHC [EnVision] 100×) **(F)** HepPar-1 positive (IHC [EnVision] 100×) **(G)** MUC-1 positive (IHC [EnVision] 100×) **(H)** P53 (wild type) positive (IHC [EnVision] 100×) **(I)** ki67 positive (IHC [EnVision] 100×).

The serum AFP level was confirmed again to be 5915.27 ng/mL. The serum levels of CEA and CA19-9 were 11.48 ng/mL (normal: <5 ng/mL) and 47.51 U/mL (normal: <37 U/mL), respectively. An upper gastrointestinal endoscopy demonstrated a small intraluminal mass at the GEJ (**Figure 1A**), and a biopsy was conducted for pathology. Abdominal CT and MRI scans showed a metastatic lesion in liver segment VIII, as well as enlarged perigastric and retroperitoneal lymph nodes (**Figures 1B–E**). A percutaneous core biopsy of the liver mass was performed. HE staining and immunohistochemical (IHC) analysis suggested GEJ HAC with liver metastasis (**Figures 1G**, **2**, **3**). Serologic workup for hepatitis virus A, B, C, D, E, and HIV was negative. A ^14^C breath test for *Helicobacter pylori* was also negative. Liver function and serum IgG4 were within normal ranges.

The patient was diagnosed with GEJ HAC with liver metastasis, clinically classified as cT_2_ N_+_ M_1_ according to the eighth TNM classification system (13).

### 2.2 Treatment

A multidisciplinary team discussion recommended comprehensive systematic treatment, rather than surgery, for this patient. The treatment regimen included chemotherapy (SOX or S-1) plus sintilimab. SOX consists of oxaliplatin (130 mg/m²) administered on day 1, and S-1 (40 mg/m² taken orally twice daily) from day 1 to day 14. Sintilimab (200 mg) was intravenously administered on day 1.

**Figure 4:**
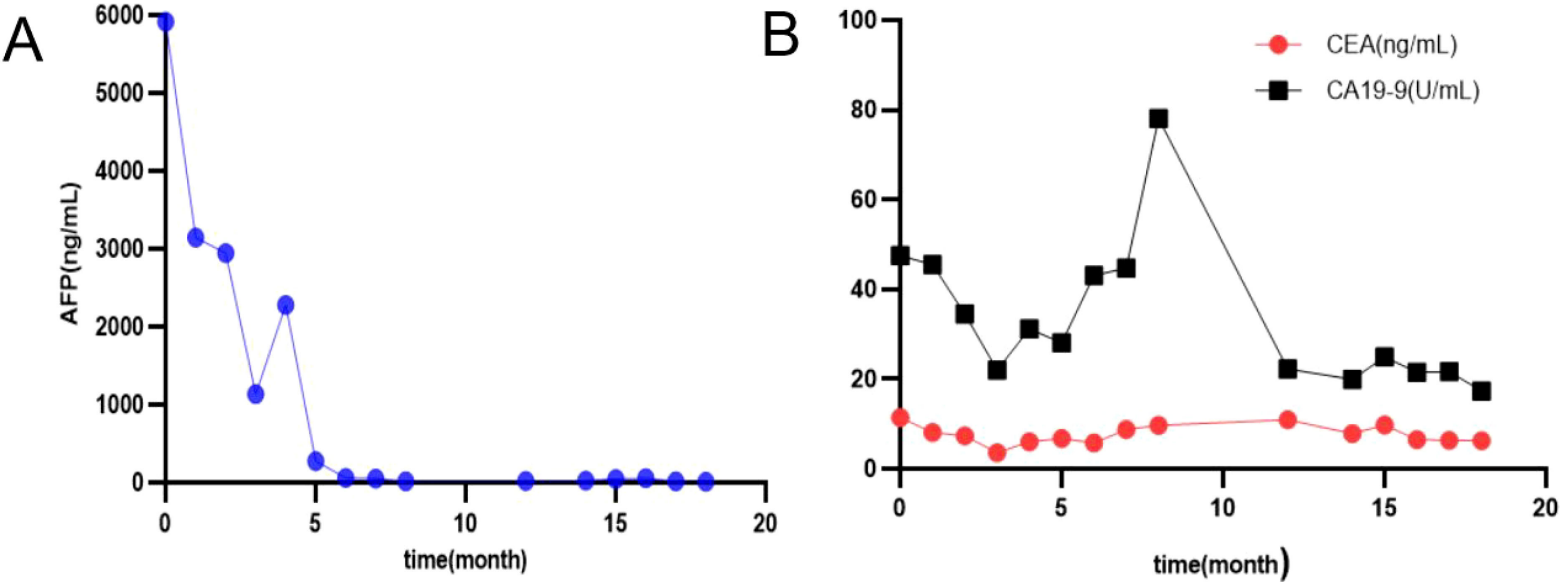
Changes in serum levels of AFP **(A)**, CA 19-9, CEA **(B)**; AFP, alfa-fetoprotein, CEA, carcinoembryonic antigen; CA 19-9, carbohydrate antigen 19-9.

After 8 cycles of treatment with SOX combined with Sintilimab, the levels of serum CEA, CA19-9, and AFP significantly decreased (**Figure 4**), and the metastatic lesion in liver segment VIII significantly shrunk (**Figures 1I, K**). From December 8, 2023, to July 7, 2024, a wait-and-watch strategy was adopted.

**Figure 5:**
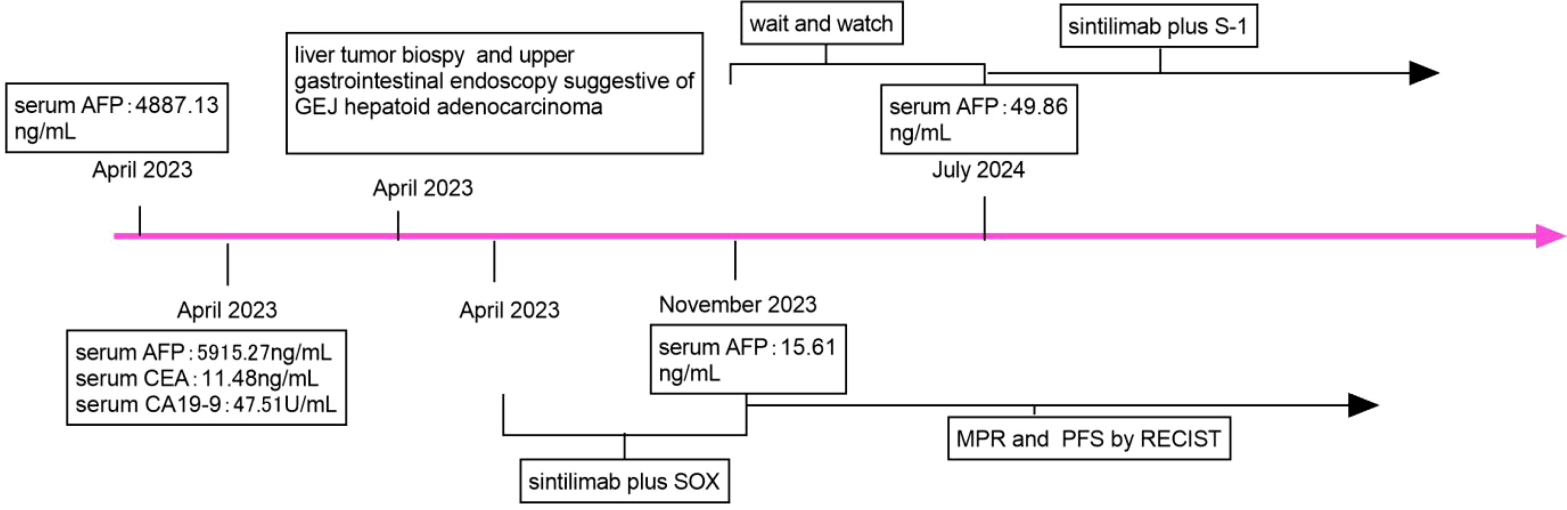
Timeline of the case report. SOX, oxaliplatin and S-1; AFP, alfa-fetoprotein; CEA, carcinoembryonic antigen; CA 19-9, carbohydrate antigen 19-9; MPR, major pathologic response; PFS, progression-free; RECIST, Response Evaluation Criteria in Solid Tumours.

For an increase in the level of serum AFP, sintilimab plus S-1 was administered as maintenance therapy until disease progression, unacceptable toxicity occurred, or for up to 24 months (**Figures 4A**, **5**).

### 2.3 Specimen processing

The tissue specimens were first fixed in 10% neutral formaldehyde and then underwent standard sampling procedures. Subsequently, they were dehydrated and embedded in paraffin, followed by sectioning to a precise thickness of 4 micrometers. These sections were stained using both traditional hematoxylin and eosin (HE) staining and immunohistochemical (IHC) testing methods. Imaging analysis was conducted using brightfield microscopy.

The antibodies used for both diagnostic and differential diagnostic purposes were purchased from Beijing Zhong Shan Golden Bridge Biological Technology Co., Ltd. The IHC testing was performed using the EnVision two-step method, with strict adherence to the kit’s instructions. Positive and negative controls were included throughout the entire staining procedure to ensure accuracy and reliability.

## 3 Results

### 3.1 Changes of tumor biomarkers

After treatment with Sintilimab-based chemotherapy (SOX or S-1), the levels of serum AFP, CEA, and CA19-9 decreased sharply (**Figure 4**).

### 3.2 Clinicopathological features

The tumor cells in the gastroesophageal junction and liver tissue exhibit a glandular shape with a nest-like adenoid structure, abundant cytoplasm, large and deeply stained nuclei, and pathologic mitosis (**Figures 1F, G**).

### 3.3 Immunohistochemistry

The IHC marker results for GEJ and metastatic liver tissue support a diagnosis of moderately to poorly differentiated hepatoid adenocarcinoma. As depicted in **Figures 2**, **3** and **Table 2**, IHC revealed that AFP, CK19,CK20, HepPar-1, MUC-1, and wild-type P53 were positive in both the GEJ mass and metastatic liver tissue. CD10 was negative and CK7 was positive for the GEJ mass, whereas CD10 was positive and CK7 was negative for metastatic liver tissue.

**Table 2:** Different IHC biomarker expression between GEJ mass and metastatic liver tissue.

	AFP	CD10	CK19	CK20	HepPar-1	Ki67	P53 (wild type)	CK7	Her-2	Arginase-1	Glypican-3	CA19-9	MUC-1
GEJ mass	+	–	+	+	+		+	+	–	–	+	+	+
metastatic liver tissue	+	+	+	+	+	+	+	–					+

### 3.4 Next-generation sequencing assay

The NGS assay of GEJ mass indicated that the *JAK2* and *TP53* genes harbored missense mutations, while the *MLH1, MSH2, MSH6, PMS2, ERBB2, EGFR, PIK3CA, APC, CTNNB1, CDH1, and DPYD* genes were normal.

## 4 Discussion

HAC, an extrahepatic tumor morphologically similar to hepatocellular carcinoma, originates from multiple organs such as the stomach, colon, esophagus, papilla of Vater, lungs, peritoneal cavity, gallbladder, adrenal gland, kidney, urinary bladder, uterus, and vagina, and frequently metastasizes to the liver and lungs (2, 14). Its symptoms are usually atypical and include abdominal pain, hematemesis, melena, fatigue, diarrhea, anorexia, nausea, obstructive jaundice, weight loss, and loss of appetite (14). As illustrated in **Table 1**, Patients with GEJ HAC are on average 54.6 years old, 92.3% of them are male, and their AFP level in serum is elevated (3–10). The positivity for GEJ HAC may serve as a possible source for the elevation of AFP levels in serum (10). In this study, the patient with GEJ HAC metastasizing to the liver was a 65-year-old man with no symptoms, and his serum AFP level was 5915.27 ng/mL.

Regarding histopathological features, HAC can be classified into two distinct types: the medullary type, characterized by polygonal cells arranged in compact nests or sheets, with scattered large, pleomorphic cells or multinucleated giant cells; and the well-differentiated papillary or tubular type, characterized by clear cytoplasm. In this study, HE staining revealed that the tumor cells in both the GEJ and liver metastasis exhibited a glandular morphology, characterized by a nest-like adenoid structure.

Regarding immunohistochemical markers, AFP, glypican-3, HepPar-1, and Sal-like protein 4 have been identified as useful in the immunohistochemical diagnosis of HAC (14). CK7 and CK19 are markers of bile duct epithelium. In this case, the positive expression of HepPar-1, AFP, glypican-3 and CK7/CK19 is in agreement with the features of hepatocellular carcinoma.

Based on findings in the histology and IHC staining, the patient was diagnosed with HAC of the GEJ with liver metastasis. In the meantime, GEJ HAC needs to be differentiated from the following tumors:

1. Primary hepatocellular carcinoma: A patient generally has a history of hepatitis and cirrhosis. An abdominal enhanced CT scan shows a mass in the liver with the characteristic of rapid in-and-out imaging. The serum AFP level is increased, and AFP is positive in IHC staining. HE staining exhibits no papillary structures. In this study, CK7 is positive for the GEJ mass but negative for liver metastasis, which supports the diagnosis of GEJ HAC with liver metastasis rather than primary HCC.
2. Germ cell tumors with liver metastasis: In yolk sac tumors and embryonal carcinomas, plasma AFP is increased, and IHC staining is positive for CD30, ER, and PR (15, 16). Neither yolk sac-like structures nor testicular masses were observed in this case.
3. GEJ carcinoma with liver metastasis: An upper gastrointestinal endoscopy reveals a lesion at the GEJ. An abdominal enhanced CT scan demonstrates a low-density mass in the liver. Plasma AFP levels are normal, and IHC staining is negative for AFP. HE staining exhibits papillary structures.
4. Neuroendocrine carcinoma: Histology shows tumor cells ranging from small to medium in size, with indistinct cytoplasmic borders. IHC staining is positive for Synaptophysin and Chromogranin A (17).

NGS assay is very helpful in elucidating the clinicopathological and molecular characteristics of HAC (18). *TP53* is one of the most commonly mutated genes in HAC (18). Copy number gains at 20q11.21-13.12 occur frequently in HAC, which is associated with poorer differentiation, increased vascular and nerve invasion, as well as a higher incidence of liver metastasis (18). In this study, *JAK2* and *TP53* were found to harbor missense mutations in GEJ HAC, which is a microsatellite-stable malignant tumor.

Treatment options for GEJ HAC include platinum-based chemotherapy and immunotherapy (19), as well as surgery combined with adjuvant therapy, which improves clinical outcomes (3–7). However, others believe that treatment options are limited and the prognosis remains very poor (8–10). Sintilimab combined with CapeOx is an option for the first-line treatment of patients with advanced or metastatic gastric/gastroesophageal junction adenocarcinoma (12). Serum AFP level had important prognostic implications (2). A preoperative serum AFP level ≥ 500 ng/ml was significantly associated with poorer overall survival (15). In this case, Sintilimab-based chemotherapy (SOX or S-1) was administered, resulting in a sharp decrease in serum AFP level, achievement of MPR and PFS according to Response Evaluation Criteria in Solid Tumors (**Figures 1H, J**). The patient will continue to receive supportive care along with the planned treatment, which consists of Sintilimab plus S-1 administered at 3-week intervals as maintenance therapy. This treatment will continue until disease progression is observed, unacceptable toxicity occurs, or for a maximum duration of 24 months.

## 5 Conclusion

This case, coupled with existing research literature, offers compelling evidence that positivity for AFP and HepPar-1 is highly beneficial in diagnosing GEJ HAC with liver metastasis. Furthermore, Sintilimab- based chemotherapy has proven efficacy in achieving a MPR and maintaining a progression-free state for a patient with GEJ HAC that has metastasized to the liver.

## Data Availability

The datasets presented in this study can be found in online repositories. The names of the repository/repositories and accession number(s) can be found in the article/**Supplementary Material**.
