# Supplementary material for: Case report: Chemotherapy plus sintilimab for the treatment of gastroesophageal junction hepatoid adenocarcinoma with liver metastasis: a case study with literature review
Source: Front Immunol. 2025 Jan 27;16:1513604. doi: 10.3389/fimmu.2025.1513604 (PMC11808132; doi:10.3389/fimmu.2025.1513604)
Supplement: Supplementary file 1 [file DataSheet1.pdf]

CARE Checklist of information to include when writing a case report

CARE case report guidelines

(cc) BY-NC-ND

Topic	Item	Checklist item description	Reported on Line
Title	1	The diagnosis or intervention of primary focus followed by the words "case report"	Line1-2
Key Words	2	2 to 5 key words that identify diagnoses or interventions in this case report, including "case report"	line37-38
Abstract (no references)	3a	Introduction: What is unique about this case and what does it add to the scientific literature?	line18-22
	3b	Main symptoms and/or important clinical findings	line23-34
	3c	The main diagnoses, therapeutic interventions, and outcomes	line24-34
	3d	Conclusion—What is the main "take-away" lesson(s) from this case?	line35-36
Introduction	4	One or two paragraphs summarizing why this case is unique (**may include references**)	line39-55
Patient Information	5a	De-identified patient specific information.	line59-60
	5b	Primary concerns and symptoms of the patient.	line61-63
	5c	Medical, family, and psycho-social history including relevant genetic information	line63-67
	5d	Relevant past interventions with outcomes	line66-67
Clinical Findings	6	Describe significant physical examination (PE) and important clinical findings.	line68-79
Timeline	7	Historical and current information from this episode of care organized as a timeline	line112-113
Diagnostic Assessment	8a	Diagnostic testing (such as PE, laboratory testing, imaging, surveys).	line68-79
	8b	Diagnostic challenges (such as access to testing, financial, or cultural)	line92-103
	8c	Diagnosis (including other diagnoses considered)	line156-170
	8d	Prognosis (such as staging in oncology) where applicable	line80-82
Therapeutic Intervention	9a	Types of therapeutic intervention (such as pharmacologic, surgical, preventive, self-care)	line83-91
	9b	Administration of therapeutic intervention (such as dosage, strength, duration)	line85-86
	9c	Changes in therapeutic intervention (with rationale)	line89-91
Follow-up and Outcomes	10a	Clinician and patient-assessed outcomes (if available)	line184-185
	10b	Important follow-up diagnostic and other test results	line89
	10c	Intervention adherence and tolerability (How was this assessed?)	line184
	10d	Adverse and unanticipated events	184
Discussion	11a	A scientific discussion of the strengths AND limitations associated with this case report	line138-188
	11b	Discussion of the relevant medical literature **with references**.	line141-146
	11c	The scientific rationale for any conclusions (including assessment of possible causes)	line146-188
	11d	The primary "take-away" lessons of this case report (without references) in a one paragraph conclusion	line190-193
Patient Perspective	12	The patient should share their perspective in one to two paragraphs on the treatment(s) they received	line198-200
Informed Consent	13	Did the patient give informed consent? Please provide if requested	Yes ☑ No ☐
